# Laparoscopic ventral mesh rectopexy *vs*. transperineal mesh repair for obstructed defecation syndrome associated with rectocele: comparison of selectively distributed patients

**DOI:** 10.1186/s12893-023-02206-0

**Published:** 2023-11-24

**Authors:** Bengi Balci, Sezai Leventoglu, Igbal Osmanov, Beyza Erkan, Yasemin Irkilata, Bulent Mentes

**Affiliations:** 1 Department of Surgery, Memorial Ankara Hospital, Proctology Unit, Ankara, Turkey; 2 Department of Surgery, Faculty of Medicine, Gazi University Hospital, Ankara, Turkey; 3 Pelvic Floor Diseases Rehabilitation Clinic, Ankara, Turkey

**Keywords:** Obstructed defecation syndrome, Laparoscopic ventral mesh rectopexy, Transperineal mesh repair, Initial Measurement of Patient-Reported Pelvic Floor Complaints Tool

## Abstract

**Purpose:**

Obstructed defecation syndrome represents 50–60% of patients with symptoms of constipation. We aimed to compare the two frequently performed surgical methods, laparoscopic ventral mesh rectopexy and transperineal mesh repair, for this condition in terms of functional and surgical outcomes.

**Methods:**

This study is a retrospective review of 131 female patients who were diagnosed with obstructed defecation syndrome, attributed to rectocele with or without rectal intussusception, enterocele, hysterocele or cystocele, and who underwent either laparoscopic ventral mesh rectopexy or transperineal mesh repair. Patients were evaluated for surgical outcomes based on the operative time, the length of hospital stay, operative complications, using prospectively designed charts. Functional outcome was assessed by using the Initial Measurement of Patient-Reported Pelvic Floor Complaints Tool.

**Results:**

Fifty-one patients diagnosed with complex rectocele underwent laparoscopic ventral mesh rectopexy, and 80 patients diagnosed with simple rectocele underwent transperineal mesh repair. Mean age was found to be 50.35 ± 13.51 years, and mean parity 2.14 ± 1.47. Obstructed defecation symptoms significantly improved in both study groups, as measured by the Colorectal Anal Distress Inventory, Constipation Severity Instrument and Patient Assessment of Constipation-Symptoms scores. Minor postoperative complications including wound dehiscence (*n* = 3) and wound infection (*n* = 2) occurred in the transperineal mesh repair group.

**Conclusion:**

Laparoscopic ventral mesh rectopexy and transperineal mesh repair are efficient and comparable techniques in terms of improvement in constipation symptoms related to obstructed defecation syndrome. A selective distribution of patients with or without multicompartmental prolapse to one of the treatment arms might be the preferred strategy.

**Supplementary Information:**

The online version contains supplementary material available at 10.1186/s12893-023-02206-0.

## Introduction

Obstructed defecation syndrome (ODS) is a disabling condition with major impact on quality of life. It was first described in 1978 as *outlet obstruction*, and eventually, it was included in the ROME IV criteria under the *functional anorectal disorders* section [1, 2]. Several underlying pathologies may cause ODS, including rectocele, rectal intussusception, pelvic floor dyssynergia, or slow-transit time constipation [3]. Patients with ODS usually suffer from straining, constipation, incomplete evacuation, and/or digitation to evacuate, with resultant changes in lifestyle [4].

Being a leading cause of ODS, rectocele is defined as a protrusion of the anterior rectal wall through the rectovaginal (RV) septum into the posterior vaginal wall, with well-known risk factors of elder age and multiparity [5, 6]. Besides, it is occasionally part of a multi-compartmental pelvic organ prolapse (PoP) associated with enterocele, cystocele or hysterocele, as also referred as *complex* rectocele [7]. Biofeedback therapy and dietary changes are the basis of the first-line treatment; however, surgery is indicated when these methods fail [8, 9].

Surgical treatment, whether transabdominal or transperineal/transvaginal/transanal, is based on the correction of rectal anatomy and function of the RV septum. Laparoscopic ventral mesh rectopexy (LVMR) has been reported to be a safe and feasible method for complex rectocele, and it has the advantage of correcting prolapse of other pelvic organs [7, 10]. Nonetheless, for patients with simple (predominant, isolated) rectocele, transperineal mesh repair (TPMR) has become our treatment of choice, with significant improvement in obstructed defecation symptoms [11].

In the present study, patients with ODS were stratified according to the diagnosis of complex or isolated, simple rectocele and designated for either LVMR or TPMR technique. The success of the two treatment methods with regard to ODS was targeted as the end point.

## Material and methods

### Study design and inclusion criteria

This study investigated a cohort of female patients who underwent LVMR or TPMR between May 2015 and July 2021 for ODS. This study is a retrospective review of patients’ charts by a prospective evaluation using validated questionnaires. The study was performed in Memorial Ankara Hospital, and it was approved by the Ethics Committee (10.06.2021/02). The study was reported according to the STROBE guidelines [12].

Patients were recruited among those who had ODS and failed with conservative management, including dietary change and biofeedback therapy. Female patients were deemed eligible for inclusion if they had anterior rectocele of Baden-Walker stages II-III [13], larger than 3 cm in size, and with one or more of the following symptoms: excessive straining, incomplete evacuation, the need for digitalization during defecation, and/or dyspareunia. Those who had a history of colorectal resection or rectocele repair, slow-transit time constipation, overt rectal prolapse, nonrelaxing puborectalis, sphincter injury, or inflammatory bowel diseases were excluded. The study investigated the outcomes in selectively distributed patient cohorts with clearly stated inclusion criteria.

In addition to colonoscopy, dynamic MRI defecography was performed in all patients to confirm the diagnosis and to reveal any concurrent pathology. Patients with a complex rectocele, which was defined as a rectocele above 3 cm with at least one additional pathology of rectal intussusception, enterocele, hysterocele or cystocele, underwent LVMR. On the other hand, TPMR was scheduled for the patients with a simple rectocele without any aforementioned pathology on MRI. Of the 144 patients who met the eligibility criteria, five patients in LVMR group and eight patients in TPMR group were lost follow-up. Two patients with complex rectocele were not suitable for the laparoscopic technique (chronic obstructive pulmonary disease and previous multiple abdominal operations), and they underwent combined perineal procedures with TPMR.

### Study variables

Clinical features including the American Society of Anesthesiologists (ASA) classification [14], body mass index (BMI), parity, operative time, intraoperative and postoperative complications, and the length of hospital stay were noted. The postoperative complications were graded according to Clavien-Dindo classification, and grade III-IV-V complications were considered as major complications [15].

Initial Measurement of Patient-Reported Pelvic Floor Complaints Tool (IMPACT) is a combination of most-used scoring systems and scales regarding the pelvic floor disorders. and it was applied for the preoperative and postoperative 12^th^ month’s functional assessment [16]. *IMPACT: bowel function assessment tool, short form* is a particular domain of this tool which includes 45 questions for women and 34 questions for men, and it covers the following instruments: Bristol scale, Wexner Incontinence Score (WIS), St. Marks Incontinence Score (SMIS), Constipation Severity Instrument (CSI), Patient Assessment of Constipation-Symptoms (PAC-SYM), and Colorectal Anal Distress Inventory (CRADI).

### Operative techniques

All operations were performed by or under the supervision of two experienced coloproctologists (SL, BM).

#### Laparoscopic ventral mesh rectopexy (LVMR)

The LVMR procedure was performed similar to the original technique described by D'Hoore [17] (Online resource 1). Bowel preparation with polyethylene glycol was done one-day prior to surgery. The patient was positioned in a Lloyd-Davies position under general anesthesia. Four trocars were inserted as follows: two 5 mm trocars from the left and right upper quadrants and two 10 mm trocars from the umbilicus and the right lower quadrant. The dissection was initiated with the opening of the pelvic peritoneum at the level of sacral promontory, and it was carried out to the pelvis with a reversed J-shaped form. The dissection was kept limited to the anterior of the rectum, and lateral or posterior dissection was avoided. Denonvillier's fascia was opened, and the dissection was continued until the pelvic floor muscles were exposed. The level of pelvic floor muscles was also confirmed with digital vaginal and rectal examination, intraoperatively. A polypropylene/polyglactin 910 mesh (Vypro®, Johnson & Johnson Ethicon Inc., USA) of 3 × 15 cm was fashioned with a distal width of 3 cm tapering it to a proximal width of 2 cm to resemble a spatula. The mesh was placed anteriorly to the rectum and fixated by two 3/0 polydioxanone sutures (PDS II®, Johnson & Johnson Ethicon Inc., USA) on each side of the rectum. Then, an adhesive material (LiquiBand®FIX8™, Advanced Medical Solution, Plymouth, UK) was used to secure the mesh to the anterior wall of the rectum. Traction was applied, and then the upper edge of the mesh was fixated to the sacral promontory by titanium tacks (ProTack™ 5 mm, Covidien, Minneapolis, MN). The pelvic peritoneum was approximated with continuous 3/0 V-Loc ™ (Medtronic Covidien, USA) sutures to cover the mesh. Pelvic drains were not placed in any patient.

#### Transperineal mesh repair (TPMR)

Spinal anesthesia was used for the majority of patients. In the lithotomy position, 15 ml of 1:2000 adrenaline solution was injected into the RV septum behind the posterior vaginal wall (Online resource 2). A transverse perineal incision was made, and a plane between the posterior wall of the vagina and the anterior wall of the rectum was developed up to the posterior fornix, using the cut mode of the electrocautery. A polyglycolic acid mesh (Soft PGA Felt®, Aventis Behring GmbH, Germany), measuring approximately 8 × 4 cm, was tailored to fit on the defect. PGA mesh soaks blood and rapidly adheres to the tissues. No sutures or additional interventions were used. Only the skin was approximated in an interrupted fashion with 3/0 polyglactin 910 sutures (Vicryl®, Johnson & Johnson Ethicon Inc., USA).

### Follow-up

Patients were followed up in the outpatient clinic at postoperative 1 and 3 months for any complications, and at 12 months for the study parameters by residents who were blinded to the study design. The anorectal and vaginal examinations were performed on each visit and the findings were recorded. The anatomical recurrence was defined as the failure of RV septum more than Baden-Walker stage I. The patients were also evaluated by using IMPACT at the postoperative 12^th^ month.

### Study endpoints

The primary endpoint was to compare these two procedures regarding treatment of ODS using IMPACT and anatomic healing. The secondary endpoints were differences in surgical outcomes, such as the operative time, operative bleeding, postoperative complications, and length of hospital stay.

### Statistical analysis

The statistical software package SPSS 22.0 for Windows® (SPSS Inc., Chicago, IL) was used. Continuous variables were reported as mean with standard deviation if normally distributed, or median with range if abnormally distributed. Categorical variables were presented as frequencies with percentages. Continuous variables were compared using the Chi-square test. The comparative analysis of two study groups regarding IMPACT scores was done using the Kolmogorov–Smirnov test. The differences between preoperative and postoperative 12^th^ month IMPACT scores within each group were analyzed using the Wilcoxon matched-pair signed-rank test. A *p*-value of < 0.05 was accepted as significant.

## Results

A total of 131 female patients were included in the final analysis: 51 patients in the LVMR group and 80 patients in the TPMR group. Patients' clinical features are shown in Table 1. The leading symptoms declared by the patients were constipation (*n* = 68), straining (*n* = 30), feeling of incomplete evacuation (*n* = 15), rectal pain (*n* = 12), fecal incontinence (*n* = 6), although most patients complained from more than one.

**Table 1:** Patients' clinical features according to the study groups

**Variables**	**Study cohort** **(*n* = 131)** Mean ± SD or Median (Min–Max) or N(%)	**LVMR** **(*n* = 51)** Mean ± SD or Median (Min–Max) or N(%)	**TPMR** **(*n* = 80)** Mean ± SD or Median (Min–Max) or N(%)	p-value*
**Age**	50.35 ± 13.51 (24–87)	48.63 ± 11.41	51.45 ± 14.66	0.25
**ASA**				
I	83 (63.35)	34 (66.66)	49 (61.25)	0.67
II	44 (33.58)	15 (29.41)	29 (36.25)	
III	4 (3.05)	2 (3.92)	2 (2.5)	
**BMI (kg/m**^**2**^**)**	24.96 ± 4.78 (18–40)	23.58 ± 4.57	25.85 ± 4.72	0.21
**Parity**	2 (0–10)	2 (0–4)	2 (0–10)	0.09
**Operative time** **(minutes)**	111.98 ± 42.77 (40–200)	151.37 ± 31.54	86.87 ± 27.21	**0.000**
**Operative bleeding (ml)**	6.62 ± 2.97 (3–20)	5.41 ± 2.06	7.40 ± 3.20	**0.006**
**Clavien-Dindo**				
**I**	3 (2.29)		3 (3.75)	**0.045**
**II**	2 (1.52)	-	2 (2.5)	
**III**	1 (0.76)		1 (1.25)	
**Mean LOS (days)**	1.10 ± 0.37 (1–4)	1.07 ± 0.27	1.12 ± 0.43	0.71
**Mean Follow-up (months)**	35.43 ± 17.09 (12–80)	29.01 ± 11.04	39.52 ± 18.97	0.21
**Recurrence**	3 (2.29)	2 (3.92)	1 (1.25)	0.31

*Abbreviations*: *LVMR* Laparoscopic ventral mesh rectopexy, *TPMR* Transperineal mesh repair, *SD* Standard deviation, *N* Number, *ASA* American Score of Anesthesiologists, *BMI* Body mass index, *LOS* Length of hospital stay

^*^p value was calculated with Chi-square test or Mann–Whitney U

Concomitant pathologies with rectocele found on MRI were enterocele (*n* = 9), hysterocele (*n* = 4), cystocele (*n* = 3), and rectal intussusception (*n* = 42). Additional procedures included transobturator tape (TOT), vaginal hysterectomy and/or primary cystocele repair for five patients in the LVMR group, and anterior colporrhaphy with Kelly plication and TOT for two patients in the TPMR group. The mean operative time (induction to the recovery from anesthesia) was 151.37 ± 31.54 and 86.87 ± 27.21 min in LVMR and TPMR groups, respectively (*p* = 0.000).

Minor postoperative complications occurred in five patients in the TPMR group: wound dehiscence in three patients and wound infection in two patients. A single major complication occurred and required surgical hemostasis on postoperative day-2 due to bleeding in the operative field. The mean length of hospital stay was 1.07 ± 0.27 and 1.12 ± 0.43 days in LVMR and TPMR groups, respectively (*p* = 0.71). Anatomical recurrence was detected in three patients (LVMR; *n* = 2 (3.92%), and TPMR; *n* = 1 (1.25%) during the mean follow-up of 35.43 ± 17.09 months.

**Fig. 1:**
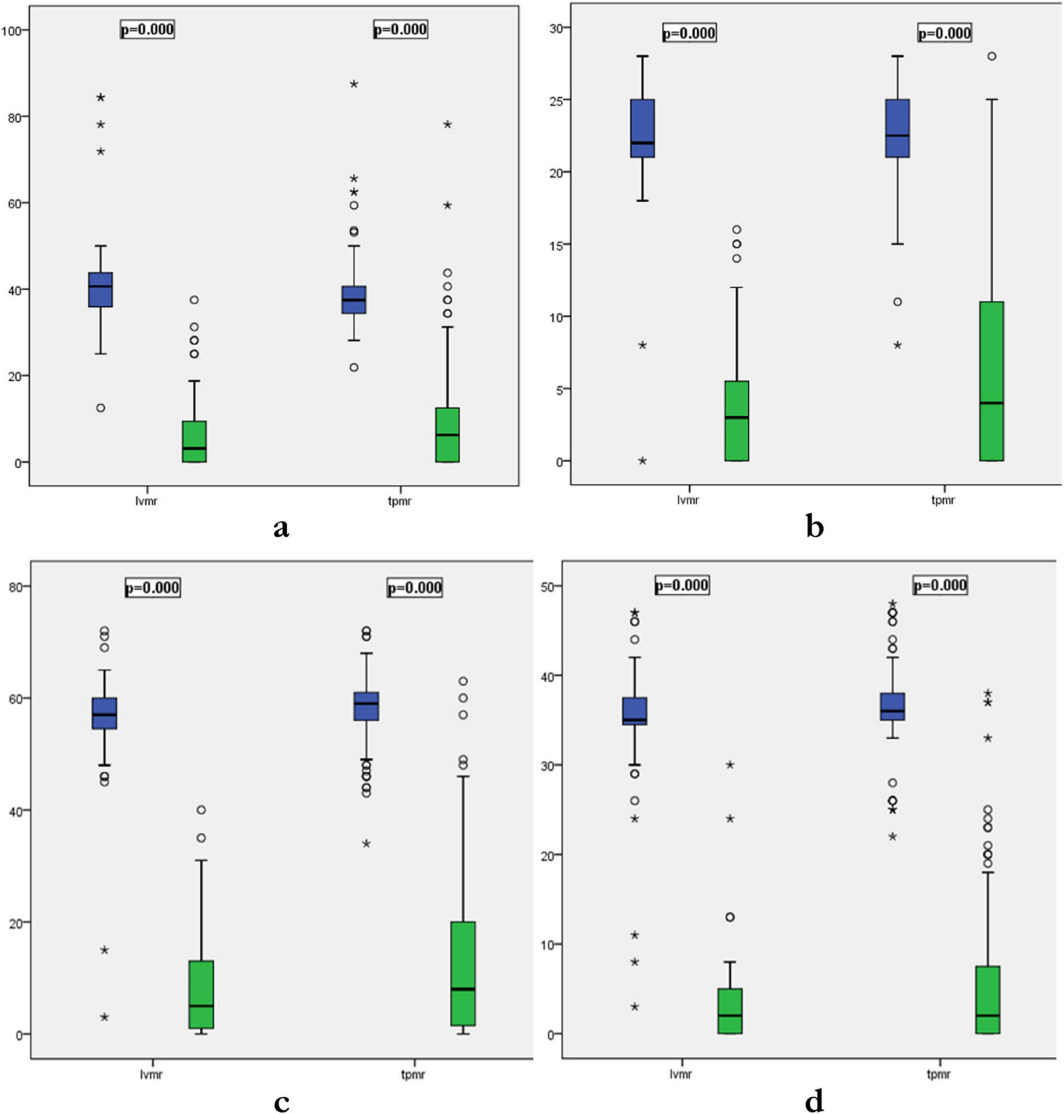
Differences between preoperative and postoperative 12^th^ month median IMPACT scores within each group: CRADI-8 scores are presented in (**a**), CSI-ODS Subscale scores in (**b**), CSI total scores in (**c**), and PAC-SYM total scores in (**d**)*.* Note that p values reflect the differences in preoperative and postoperative CRADI-8, CSI-ODS and CSI scores within each group and the comparisons of outcomes between study group are presented in Table 2. *p value* was calculated with Wilcoxon matched-pair signed-rank test

The comparative analysis of preoperative and postoperative 12^th^ month median IMPACT scores between the LVMR and TPMR groups revealed no differences, except for the preoperative CSI-pain subscale (Table 2). Further analysis within each group demonstrated statistically significant differences between the preoperative and postoperative 12^th^ month median CRADI-8, CSI total, and PAC-SYM total scores as shown in boxplots (Fig. 1). The decreasing trend in postoperative Wexner and SMIS scores did not reach statistical significance in either group.

**Table 2:** Comparison of preoperative and postoperative IMPACT scores in LVMR and TPMR groups

**Variables**	**LVMR** **(*n* = 51) Median (Min–max)**	**TPMR** **(*n* = 80) Median (Min–max)**	p-value*	**LVMR** **(*n* = 51) Median (Min–max)**	**TPMR** **(*n* = 80) Median (Min–max)**	p-value*
**IMPACT scores**	***Preoperative***			***Postoperative 12***^***th***^*** month***		
WIS	0 (0–19)	0 (0–20)	0.99	0 (0–15)	0 (0–20)	1.00
SMIS	0 (0–23)	0 (0–22)	0.99	0 (0–17)	0 (0–22)	0.93
CRADI-8	40.62 (13–84)	37.5 (22–88)	0.78	3.12 (0–38)	6.25 (0–78)	0.49
CSI Total score	57 (3–72)	59 (34–72)	0.96	5 (0–40)	8 (0–63)	0.08
CSI- ODS Subscale	22 (0–28)	22.5 (8–28)	1.00	3 (0–16)	4 (0–28)	0.13
CSI-Colonic Inertia Subscale	23 (1–29)	23 (15–29)	0.99	2 (0–20)	5 (0–26)	0.14
CSI- Pain Subscale	11 (2–16)	12 (3–16)	**0.01**	0 (0–7)	0 (0–12)	0.20
PAC-SYM Total score	35 (3–47)	36 (22–48)	0.09	2 (0–30)	2 (0–38)	0.30

*Abbreviations*: *LVMR* Laparoscopic ventral mesh rectopexy, *TPMR* Transperineal mesh repair, *SD* Standard deviation, *WIS* Wexner Incontinence Score, *SMIS* St. Marks Incontinence Score, *CRADI-8* Colorectal Anal Distress Inventory, *CSI* Constipation Severity Instrument, *PAC-SYM* Patient Assessment of Constipation-Symptoms

^*^p value was calculated with Indepent Kolmogorov–Smirnov (2 samples) test

The number of patients who had an improvement over 50% at postoperative 12^th^ month’s IMPACT scores were shown for each group in Table 3. In the LVMR group, 49 patients (96%) had statistically significant CRADI-8 (*p* = 0.021) and PAC-SYM (*p* = 0.031) improvements over 50% at postoperative 12^th^ month.

**Table 3:** Number of patients with an improvement over 50% at postoperative 12^th^ month’s IMPACT scores

Variables	Patients’ improvement N (%)		*p-value*
**IMPACT scores**	**LVMR** **(*n* = 51)**	**TPMR** **(*n* = 80)**	
WIS	4 (36)	3 (25)	0.55
SMIS	4 (33)	2 (16)	0.34
CRADI-8	49 (96)	66 (82)	**0.021**
CSI Total score	46 (90)	63 (78)	0.08
CSI-ODS Subscale	44 (86)	60 (75)	0.12
CSI-Colonic Inertia Subscale	43 (84)	60 (75)	0.20
CSI-Pain Subscale	50 (98)	73 (91)	0.11
PAC-SYM Total score	49 (96)	67 (83)	**0.031**

*Abbreviations*: *LVMR* Laparoscopic ventral mesh rectopexy, *TPMR* Transperineal mesh repair, *SD* Standard deviation, *WIS* Wexner Incontinence Score, *SMIS* St. Marks Incontinence Score, *CRADI-8* Colorectal Anal Distress Inventory, *CSI* Constipation Severity Instrument, *PAC-SYM* Patient Assessment of Constipation-Symptoms

The re-assessment of patients with continuing symptoms revealed failure of the RV septum in 3 patients and re-operation was offered. Biofeedback therapy and dietary changes with supplements were recommended for patients with obstructed defecation symptoms without any detectable anatomic failure.

## Discussion

The present study demonstrates that LVMR and TPMR are efficient and comparable techniques in reconstructing the defective RV septum and alleviating constipation symptoms related to ODS. This is one of the few studies comparing a perineal approach with an abdominal/laparoscopic one for the treatment of ODS due to rectocele. Besides, the functional outcome was evaluated by the comprehensive tool IMPACT. The study, by definition, is not based on a randomized comparison, and it examines and assesses the outcomes in selectively distributed patient cohorts with clearly stated inclusion criteria.

We reasonably treated our patients with *complex* rectocele with LVMR. Not infrequently, rectocele is part of a more complex PoP, associated with enterocele, cystocele, hysterocele, and/or rectal intussusception or rectal prolapse. In such a case, repairing only the rectocele by transperineal, transanal, or transvaginal route would inevitably be an inadequate procedure. On the other part, LVMR, as a modern approach, has its own limitations, and an ideal treatment algorithm for ODS due to rectocele is still debatable. Our results might provide further evidence for the developing algorithm for the treatment of ODS due to rectocele. The selective distribution of the patients to groups such as LVMR, TPMR, biofeedback, and excluded cases with additional pathologies results in modest number of cases in some groups with potential impact on sample size.

LVMR has been commonly preferred in patients presented with ODS in case of rectal intussusception with or without enterocele and rectocele [18]. We obtained good anatomical and functional results with LMVR in our selected group of patients with *complex* rectocele that support findings from previous studies [19–21]. The perineal descent, thus overall pelvic floor function only improves after ventral rectopexy, presumably as the result of additional pulling effect on the levator muscles. Moreover, better long-term functional outcomes with LVMR compared to perineal approaches is one of the advantages of this technique [22–24]. Similarly, we have found that more patients had an improvement in CRADI-8 and PAC-SYM scores over 50% at postoperative 12^th^ month in the LVMR group. This result might be attributed to more lasting effects of ventral rectopexy over transperineal repair, which is similar to comparative outcomes of this technique over STARR (stapled transanal rectal resection) in long-term [23].

It’s also remarkable that we experienced no major complications after LVMR. However, the operation requires, anterior dissection of the rectum down to the levator muscles, and complications such as bladder injury have been reported [7]. Some cases may need to be converted to laparotomy because of complications or technical difficulties. A BMI > 35 and possible comorbidities in elderly patients might be additional limitations for a laparoscopic procedure. LVMR takes significantly longer than TPMR and any other anal/perineal procedures reported [7, 20]. Therefore, we need an alternative method for the treatment of rectocele even if the growing trend to and the satisfactory results with LVMR continue.

We have gained wide experience with the surgical treatment of rectocele, and a large series of us reporting the results of TPMR for rectocele was reported in 2007 [11]. Compared with this pervious series of ours, our presents results are even better, possibly due to better patient selection and experience. Our results revealed that in selected patients, TPMR compares favorably with LVMR to treat ODS due to rectocele. Although we work from two completely different perspectives, we restore the defective RV septum successfully. As suggested by Watson and coworkers, mesh repair of rectocele deals with the cause (failure of the rectovaginal septum) rather than the effect [25]. The PGA mesh is easy to handle, and it helps surgical hemostasis in a highly vascularized tissue plane, also it attracts strong connective tissue rapidly to the defective RV septum. We’ve never encountered mesh reaction or mesh protrusion with the PGA mesh although we are well aware of the dramatic cases of mesh reaction and the timidity of surgeons to use a mesh, especially within the pelvis.

An alternative approach to TPMR is a transvaginal repair. Ferrari et al. reported global improvement of symptoms in 87.9% of patients, while ODS-related symptoms improved in only 58% [26]. A randomized-controlled trial on transperineal and transvaginal approaches revealed that constipation scores significantly decreased in both groups with no difference regarding recurrences [27]. Interestingly, increased resting and squeeze anal pressures were only found after transperineal repair.

Nevertheless, tailoring surgery for patients with ODS and considering the “iceberg diagram” for this unique condition have become the new rationale [28]. As well as the simplicity and complexity of the rectocele, the sphincter function is also one of the determinant factors in choosing the right technique [29]. Transperineal repair is usually preferred in patients with simple rectocele and poor sphincter function, whereas transanal and transvaginal repairs are preferred in cases with good sphincter function. On the other hand, a trend toward LVMR is seen in patients with complex rectocele regardless of the sphincter function.

The major drawback of our study appears to be non-randomized selection of patients for each procedure; however, possible co-existence of other organ prolapses, inevitably results in a selective distribution. Large number of patients with simple rectocele in our series is probably due to the selective admission of patients with ODS to our Proctology Unit, while patients with multicompartmental PoP are treated by gynecologists or urologists, too.

**Fig. 2:**
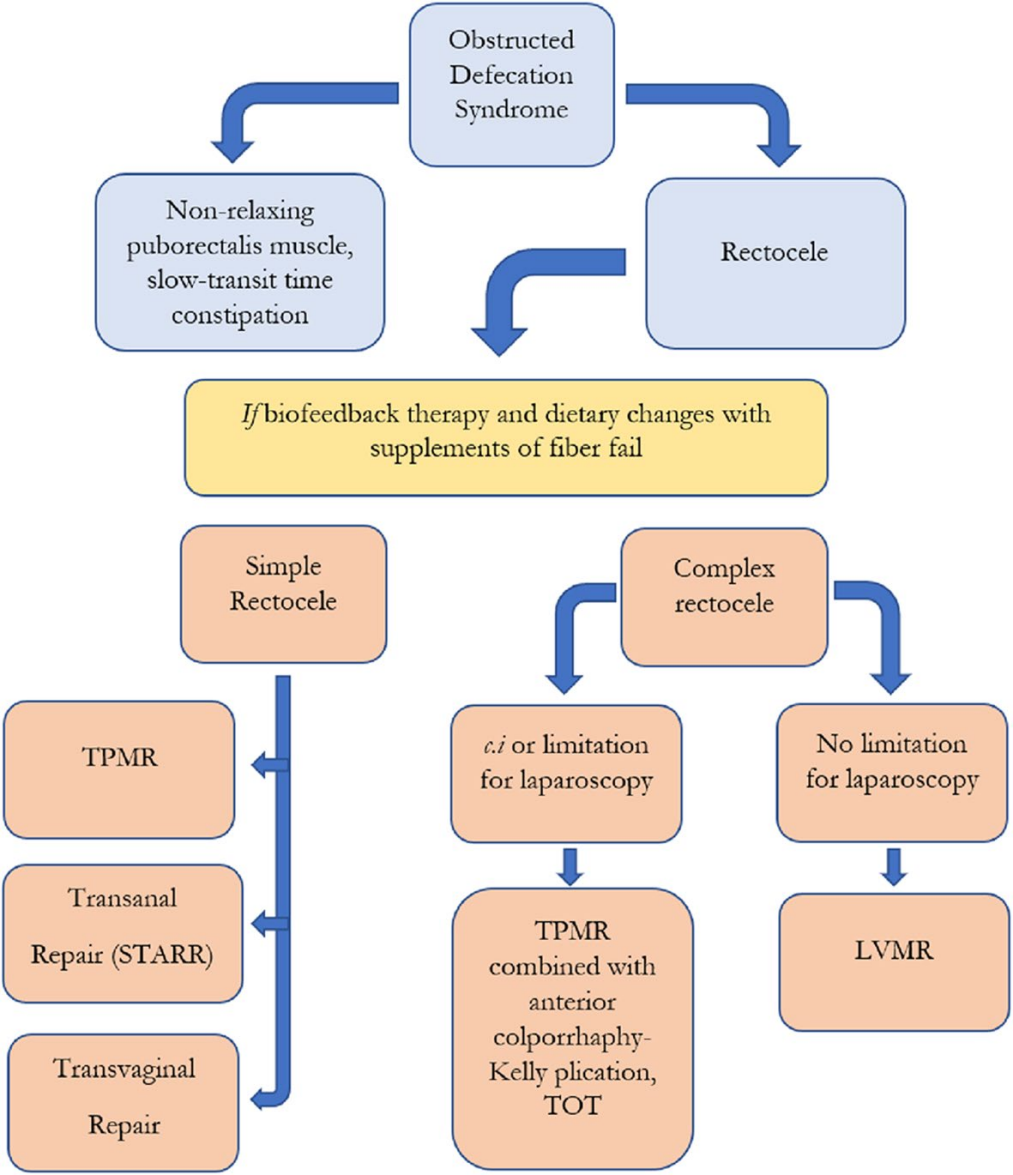
Suggested-algorithm for patients with obstructed defecation syndrome attributed to rectocele. Abbreviations: LVMR, laparoscopic ventral mesh rectopexy; TPMR, transperineal mesh repair; STARR, stapled transanal rectal resection; TOT, transobturator tape

In conclusion, our strategy is to *i)* differentiate patients with rectocele according to the presence or absence of other compartmental prolapse, *ii)* treating complex rectocele with LVMR unless technical difficulties or contraindications for laparoscopy exist, and *iii)* treating simple rectocele with TPMR. Patients with complex rectocele and not suitable for laparoscopic technique may need TPMR + additional perineal/vaginal procedures such as anterior colporrhaphy and TOT (Fig. 2).

### Supplementary Information

**Additional file 1.**

## Data Availability

The datasets generated during and/or analyzed during the current study are available from the corresponding author on reasonable request.
